# Designing the learning of intraprofessional collaboration among medical residents

**DOI:** 10.1111/medu.14868

**Published:** 2022-07-24

**Authors:** Natasja Looman, Jacqueline de Graaf, Bart Thoonen, Dieneke van Asselt, Esther de Groot, Anneke Kramer, Nynke Scherpbier, Cornelia Fluit

**Affiliations:** ^1^ Department of Primary and Community Care Radboudumc Nijmegen The Netherlands; ^2^ Radboudumc Health Academy Nijmegen The Netherlands; ^3^ Department of Geriatric Medicine Nijmegen The Netherlands; ^4^ Julius Center for Health Sciences and Primary Care UMC Utrecht Utrecht The Netherlands; ^5^ Department of Public health and Primary Care Leiden UMC Leiden The Netherlands; ^6^ Department of General Practice and Elderly Care University Medical Centre Groningen Groningen The Netherlands; ^7^ Department for Research in Learning and Education Radboudumc Health Academy Nijmegen The Netherlands

## Abstract

**Background:**

To preserve quality and continuity of care, collaboration between primary‐care and secondary‐care physicians is becoming increasingly important. Therefore, learning intraprofessional collaboration (intraPC) requires explicit attention during postgraduate training. Hospital placements provide opportunities for intraPC learning, but these opportunities require interventions to support and enhance such learning. Design‐Principles guide the design and development of educational activities when theory‐driven Design‐Principles are tailored into context‐sensitive Design‐Principles. The aim of this study was to develop and substantiate a set of theory‐driven and context‐sensitive Design‐Principles for intraPC learning during hospital placements.

**Methods:**

Based on our earlier research, we formulated nine theory‐driven Design‐Principles. To enrich, refine and consolidate these principles, three focus group sessions with stakeholders were conducted using a Modified Nominal Group Technique. Next, two work conferences were conducted to test the feasibility and applicability of the Design‐Principles for developing intraPC educational activities and to sharpen the principles into a final set of Design‐Principles.

**Results:**

The theoretical Design‐Principles were discussed and modified iteratively. Two new Design‐Principles were added during focus group 1, and one more Design‐Principle was added during focus group 2. The Design‐Principles were categorised into three clusters: (i) *Culture*: building collaborative relations in a psychologically safe context where patterns or feelings of power dynamics between primary and secondary care physicians can be discussed; (ii) *Connecting Contexts*: making residents and supervisors mutually understand each other's work contexts and activities; and (iii) *Making the Implicit Explicit*: having supervising teams act as role models demonstrating intraPC and continuously pursuing improvement in intraPC to make intraPC explicit. Participants were unanimous in their view that the Design‐Principles in the *Culture* cluster were prerequisites to facilitate intraPC learning.

**Conclusion:**

This study led to the development of 12 theory‐driven and context‐sensitive Design‐Principles that may guide the design of educational activities to support intraPC learning during hospital placements.

## Abstract 2

Looman et al.‘s exploration of design principles offers guidance for development of hospital placements that can enhance collaboration between primary care and specialists.

## 1 INTRODUCTION

The increasing number and complexity of patients with multimorbidity results in shifting health care system demands.^1^, ^2^, ^3^ Consequently, a growing number of patients needs to be seen by multiple physicians from primary care (e.g. family physicians in the primary care setting) and secondary care (e.g. medical specialists in the hospital setting).^4^ Meanwhile, the tendency is to provide health care for patients in a primary care setting whenever possible, leading to increased patient transitions.^5^ As both complexity and transitions in care are related to a risk of error, it is important to share knowledge and to provide coherent and coordinated care to prevent adverse events.^4^, ^6^, ^7^, ^8^, ^9^ Therefore, intraprofessional collaboration (intraPC) between primary and secondary care physicians is becoming increasingly important.^10^, ^11^, ^12^, ^13^, ^14^ There are, however, misunderstandings and paradigm conflicts between primary and secondary care physicians,^14^, ^15^, ^16^, ^17^, ^18^, ^19^ such as imbalance in authority, power conflicts, lack of knowledge of each other's roles and boundary friction when delivering patient care. These can negatively impact collaborative care and therefore negatively impact patient care and safety.^15^, ^16^ As proficient intraPC is vital to maintain quality of care,^12^, ^13^, ^15^, ^20^ and to preserve continuity of care,^7^, ^14^, ^21^, ^22^ intraPC learning requires attention.^14^, ^23^, ^24^

Previous studies have shown that primary care (PC) and secondary care (SC) residents are predominantly trained in isolation from each other and that they do not tend to build professional relations with each other due to clinical commitments, logistical challenges and curricular limitations.^25^, ^26^ A distinctive moment when PC residents and SC residents do meet is during hospital placements where PC residents work at the same hospital department as SC residents.^27^ Hospital placements are a regular element of postgraduate training programmes of PC residents and occur worldwide.^27^, ^28^, ^30^, ^31^, ^32^ Prior studies have shown that these placements provide numerous opportunities for intraPC learning^25^, ^27^, ^33^ but that these opportunities require specific interventions to support and enhance learning.^27^

To date, evidence of the characteristics and the process of designing and developing educational activities, specifically targeting intraPC learning during hospital placements, is lacking. Hospital placements are complex settings that are affected by many factors, including stakeholders from different professions with their interpersonal dynamics, different interests and delicate collaboration.^34^, ^35^, ^36^ The development of feasible and applicable intraPC educational activities in such a complex context requires a systematic approach that integrates (learning) theory and involves relevant stakeholders to align theory with local practical contexts.^36^, ^37^, ^38^, ^39^ To this end, a design‐based research approach is useful to, first, formulate theoretical Design‐Principles based on literature, and second, to enrich and align these Design‐Principles with the practice context in close collaboration among researchers and stakeholders with different areas of expertise.^35^, ^39^, ^40^ Theory‐driven and context‐sensitive Design‐Principles can serve as guidance for educational activities^37^, ^41^, ^42^ as Design‐Principles can provide prescriptive theoretical and practical understanding.^42^

This study aims to develop and substantiate both theory‐driven and context‐sensitive Design‐Principles to guide the development of intraPC educational activities during hospital placements (Box 1).

**BOX 1:** Definitions of primary and secondary care physicians

Primary care physician	A physician working in the frontline of a health care system, treating common medical problems, including physical, psychological and social prevention, cure and care. Patients have direct access to primary care physicians. Primary care physicians may play a gatekeeping role, which makes them responsible for appropriate referral of patients to hospitals and other medical services for specialised medical care.
Secondary care physician	A physician providing (planned) specialised medical care or emergency care, usually in a hospital setting. Secondary care is provided primarily on referral from another (primary care) physician.

## 2 METHODS

This study is part of a Design‐Based Research project. Characteristic of Design‐Based Research is the discovering, designing, developing and evaluating activities in a systematic and iterative way to solve complex problems in practice.^35^, ^39^, ^40^ The starting‐point for our Design‐Based Research is an educational problem for which no or only a few validated principles (guidelines or heuristics) are available to guide the design and development of educational activities. Informed by prior research and review of relevant literature, researchers in collaboration with practitioners design and develop feasible and applicable educational activities by carefully studying successive versions (or prototypes) of activities in their contexts.^35^, ^40^, ^43^ While doing so, they reflect on their research process with the purpose of producing Design‐Principles.^35^, ^40^, ^43^ Design‐Principles are typically used as heuristic guidelines to improve educational practice.^35^, ^40^

### 2.1 Design

**FIGURE 1:**
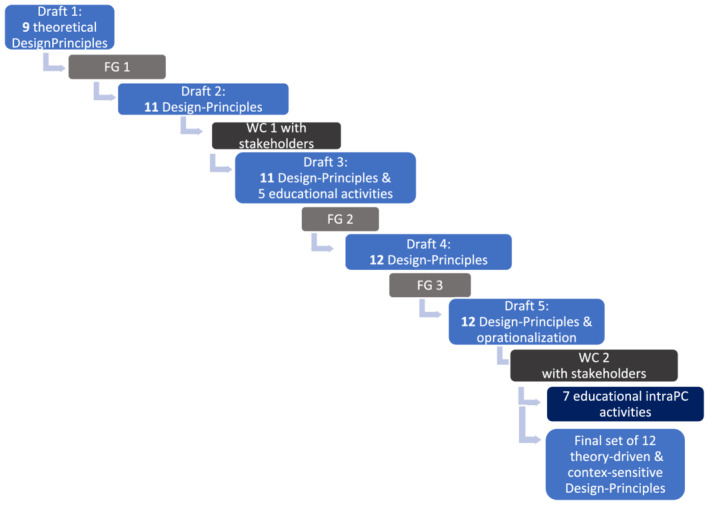
Overall process overview. FG, focus group; WC, work conference [Color figure can be viewed at wileyonlinelibrary.com]

Within Design‐Based Research, three phases can be distinguished: (I) a preliminary phase, (II) a prototyping phase and (III) an assessment phase.^44^ In the previous part of our Design‐Based Research project (phase I), we gained knowledge of what and how residents actually learn during their hospital placements and what intraPC learning improvements are needed, based on a literature review and observations and interviews with PC residents, SC residents and supervisors.^19^, ^27^ In the present study (phase II), the research group developed nine theoretical concepts of Design Principles: Design‐Principles‐Draft 1. In focus group sessions and work conferences with various stakeholders, Design‐Principles‐Draft 1 was enriched and consolidated into a final set of validated theory‐driven and context‐sensitive Design‐Principles. An overview of this process is shown in Figure 1. The third, assessment, phase is outside the scope of this paper.

**FIGURE 2:**
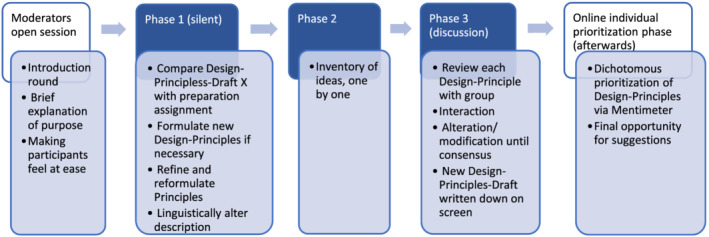
Process overview focus group sessions with modified Nominal Group Technique (NGT) structure [Color figure can be viewed at wileyonlinelibrary.com]

We considered an iterative process of focus groups and work conferences an appropriate method for capturing the ideas, perceptions, feelings and circumstances of stakeholders.^45^ We used focus group sessions with a Modified Nominal Group Technique (NGT)^46^ to discuss, enrich, refine and consolidate Design‐Principles. NGT makes use of a prioritising process. Variations to this prioritisation process are often used in research to fit the purpose and setting of a specific study, which is called a modified NGT.^47^, ^48^, ^49^, ^50^ We chose individual online prioritisation of the design principles after finishing the third focus group session.^51^, ^52^, ^53^, ^54^, ^55^, ^56^ We performed multiple focus group sessions with a combination of the modified NGT method as the one described by Seidel and the one described by Søndergaard^49^, ^50^ (see Figure 2).

Additionally, work conferences with stakeholders and patients as experts were organised to design prototypes of educational intraPC activities based on the Design‐Principles to check feasibility and applicability in practice and to further sharpen formulation of the Design‐Principles. An expert work conference has previously been described as a research method^57^ for generating creative ideas.^58^, ^59^

### 2.2 Study setting and participants

#### 2.2.1 Focus group sessions with NGT

We conducted three focus group sessions in the Netherlands. To enable direct interaction with and observation of the participants, the focus groups were led by a moderator and an observer^45^ (FG1: two psychologists (independent researcher and NL), FG2, FG3: educationalist and psychologist (CF and NL)). We included residents, medical directors, supervisors and educationalists from both primary and secondary care specialty training (see Table 1) with at least 6 months experience working at a hospital ward and/or coaching residents during hospital placements and/or teaching or investigating intraPC learning. We included six to nine participants per group.^45^, ^60^

**TABLE 1:** Participants focus group sessions and work conferences

Participant characteristics	Focus groups			Work conferences		
		Male	Female		Male	Female
Secondary Care Residents	6	(2	4)	7	(2	5)
Geriatrics	3	(1	2)	3	(2	1)
Internal medicine	1	(1	0)	‐	‐	‐
Paediatrics	1	(0	1)	2	(0	2)
Hospital physician	1	(0	1)	‐	‐	‐
Surgery	‐	‐	‐	1	(0	1)
Neurology	‐	‐	‐	1	(0	1)
Primary Care Residents	5	(0	5)	8	(1	7)
General Practitioner	2	(0	2)	4	(0	4)
Elderly care Physician	3	(0	3)	4	(1	3)
Secondary Care Supervisors	4	(0	4)	8	(1	78)
Geriatrician	2	(0	2)	3	(0	3)
Internist	1	(0	1)	1	(0	1)
Elderly care physician 2^nd^ care	1	(0	1)	‐	‐	‐
Paediatrician		‐	‐	3	(1	2)
Geriatrician–pharmacologist		‐	‐	1	(0	1)
Primary Care Teachers Supervisors	5	(0	5)	11	(3	8)
General Practitioner	4	(0	4)	7	(1	6)
Elderly care physician	1	(0	1)	4	(2	2)
Educationalists	3	(1	2)	8	(1	7)
Researchers/policy makers	‐	‐	‐	8	(1	7)
Patients/caregivers	‐	‐	‐	8	(3	8)
Total	23	(3)	(20)	58	(12)	(46)

#### 2.2.2 Work conferences

We conducted work conferences with stakeholders from the Netherlands and Belgium, which included residents, supervisors, educationalists, policy makers and researchers from primary and secondary care specialty training and patients/caregivers. The invited patients/caregivers had experience as patients or caregivers as well as experience in medical education, and so they were able to bring in the patient/caregiver's perspective in keeping with medical education. The work conferences were moderated by members of the research team and an independent educationalist.

The participants of both the focus group sessions and the work conferences were invited through the research team's network, making use of purposive sampling.^45^, ^61^ Heterogenous groups were used to gather information from different perspectives and interests across all the disciplines involved^36^, ^45^ and to avoid bias that could arise in homogeneous groups.^60^

### 2.3 Procedure

#### 2.3.1 Focus group sessions

Prior to the focus group session, we sent an information letter stating the purpose of our study together with a preparatory assignment to all participants. The assignment was to think about relevant aspects of intraPC learning experiences. For an overview of the focus group session process, see Figure 2.

The Design‐Principles (Drafts) were shown and shared on the PC screen. In phase 1 (silent phase), participants were asked to compare Design‐Principles‐Draft (1/3/4) with their preparation assignment and assess whether their outcome met any of the Design‐Principles. We asked the participants to refine, reformulate and alter the description of Design‐Principles to increase the adequacy of the Design‐Principles and possibly to formulate a new Design‐Principle if they felt this was necessary. After this phase, participants were invited to contribute their ideas to the group one by one (phase 2). Next, in phase 3, the participants reviewed and discussed each Design‐Principle and altered it until the group reached consensus about its formulation.

The Design‐Principles‐Draft outcome of the previous group was presented to the next group (see Figure 1). The researchers (CF and NL) explained the Design‐Principles‐Draft and gave a process summary of the previous focus group. The present group then re‐edited the outcome of the previous group until consensus about the formulation of Design‐Principles. Data were gathered until the last group reached a consensus about the formulation of both the Design‐Principles and the oprationalisations. As described by Kidd et al. (2000), this process can be seen as a content validation process because each group judges the credibility of outcomes derived from the previous group.^62^

Finally, Design‐Principles‐Draft 5 was sent to all focus group participants by Mentimeter© as member checking. Participants prioritised each Design‐Principle dichotomously as ‘must have’ or as ‘nice to have’, and they could comment the final set of Design‐Principles.

#### 2.3.2 Work conferences

**FIGURE 3:**
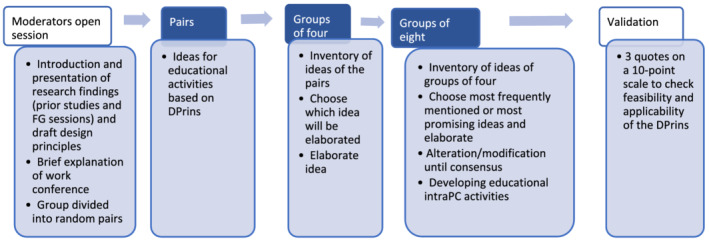
Process overview of two work conferences [Color figure can be viewed at wileyonlinelibrary.com]

At the start of each work conference, we presented the results of our previous studies and Design‐Principles‐Draft 2/5. Next, we divided the participants into pairs and asked them to create ideas for educational activities based on the Design‐Principles and think what conditions were needed for applying them. Activities might include, for example, workplace learning activities at the hospital ward, activities during release days where residents learn with colleagues from their own discipline or with intraprofessional colleagues or training activities for supervisors. Then, the participants discussed their ideas in groups of four, chose the most promising idea and elaborated this further. Finally, the ideas were discussed in groups of seven to eight until consensus was reached on the most promising idea(s). After that, the groups of seven to eight participants developed prototypes of educational activities for intraPC learning. During this process, the patients/caregivers provided feedback on the activities. For an overview of the work conference process, see Figure 3. At the end of work‐conference‐2, participants were asked to rate three quotes on a 10‐point scale to check the feasibility and applicability of the Design‐Principles. The quotes were about (i) feasibility of Design‐Principles to design intraPC educational activities; (ii) clarity of the way Design‐Principles were formulated; and (iii) applicability of Design‐Principles in real life.

### 2.4 Data analysis

Data collection and analysis occurred iteratively, and the data were discussed both between the moderator and observer and, within the research team, between the different steps of the process.^45^ An iterative process was used while building and enriching Design‐Principles. The data gathered in the individual steps (focus groups and work conferences) and throughout the whole process, functioned as a logbook to describe the process of the development of Design‐Principles, to illustrate how the Design‐Principles came about. It is common within Design‐Based Research to integrate one interim outcome into the next step of the development process.^38^ Supplemental data gathered during the focus group sessions (by audio‐recordings and transcripts) and work conferences were reread to substantiate the formulation and content of the Design‐Principles and to capture non‐verbal communication, interaction between participants and atmosphere.

### 2.5 Ethical considerations

This study was approved by the Ethical Review Board (ERB) of the Dutch Organization for Medical Education NERB dossier number: 2020.1.4. Written informed consent for the use of the audio recordings and gathered data were obtained from the participants.

## 3 RESULTS

We conducted three focus group sessions taking between 77 and 99 minutes per group with a total of 23 participants; the first was conducted live at the Radboud University Medical Center in February 2020; the second and third focus groups were conducted online via Zoom during the Covid pandemic in October and November 2020. The online prioritisation survey was completed by 20 out of 23 focus group participants in December 2020.

We conducted two work conferences (120 resp. 180 minutes per conference) with a total of 58 participants (10 resp. 48); the first was conducted live at the Radboud University Medical Center in March 2020; the second was conducted online via Zoom in February 2022. The online survey questions for validation of Design‐Principles were completed by 43 out of 48 participants in work‐conference‐2. For an overview of participants' characteristics, see Table 1.

### 3.1 Design Principles

The initially theory‐driven Design‐Principles‐Draft 1 consisted of nine Design‐Principles divided into three clusters: Design, Practical Aspects and Culture. Two new Design‐Principles (**4** and **8**) were added during focus group 1, and one more Design‐Principle (**Zero**) was added during focus group 2. The remaining Design‐Principles **1, 2, 3, 5,6, 9, 10** and **11** and the operationalisations were discussed, modified and linguistically refined in all focus group sessions and work conferences. In general, participants were in full agreement that these principles needed to be translated into their own local practices in order to make Design‐Principles applicable and appropriate to all stakeholders involved.

Our study resulted in a final set of 12 Design‐Principles for intraPC learning during hospital placements categorised into three clusters, entitled: *Culture* (Zero, 1, 2), *Connecting Contexts* (3, 4) and *Making the Implicit Explicit* (5–11) (see Table 2). The majority of Design‐Principles consisted of two parts: (i) a title, describing the design principle (the dot on the horizon) and a subtitle, describing *how* the Design‐Principle aim can be achieved; (ii) an operationalisation, describing *what* could be done to achieve the Design‐Principle aim.

**TABLE 2:** Final set of 12 Design Principles

		Design Principles
*Culture*	0	The patient is the starting‐point for working and learning
	1	Build relations with intraprofessional (primary–secondary care) colleagues PC and SC residents and supervisors invest in building equal interpersonal relations founded on mutual respect and appreciation.
		*Operationalization*: *Getting to know each other informally, building primary‐secondary care collaborative relations*. *Investing in formal primary‐secondary care collaborative relations and investing in getting to know each other's work areas*.
	2	Apply the principle that, in a intraPC partnership, we are all different but operate on a basis of equity Supervisors and PC and SC residents create a safe learning and working environment in which culture, equity and differences in work relations can be discussed
		*Operationalization*:*A safe working and learning climate (psychological safety), in which everyone feels free to raise questions or make contributions without this having any negative consequences*. *‘(Learning how to) collaborate intraprofessionally’ on the basis of equality and respect*. *Recognising historical patterns and feelings of differences in power and culture and opening these up for discussion*.
Connecting Contexts	3	Facilitate learning together by working together Those responsible for curricula ensure that the *physical* workplaces and work schedules facilitate *daily collaboration* and mutual learning between PC and SC residents.
		*Operationalization*:*Facilities: physical time and space for encounters*. *Create time and space for supervision and team reflection and joint education*.
	4	Facilitate the acquisition of knowledge of one another's work contexts and activities to promote good collaboration. Those responsible for training programmes facilitate residents in getting to know each other's contexts, interests, needs, (im)possibilities, activities and necessities so as to improve collaboration for quality care
		*Operationalization*:*For example by having SC residents do placements in primary care*.
Making the implicit explicit	5	Collaborate on patients and pay deliberate attention to two‐way learning from different perspectives.Supervisors, teachers and residents make sure that joint workplace learning places the patient at the centre as seen from each other's (PC and SC) perspectives and curiosity. Supervisors, teachers, designers and residents make sure that form and content do justice to the perspectives and the expertise of both PC and SC residents and supervisors.
		*Operationalization*: *Proactive two‐way learning and making intraPC learning explicit*. *PC residents contribute their own experience and knowledge to secondary care*.
	6	Purposely discuss intraPC collaboration during daily work activities. Residents and supervisors utilise everyday work meetings and patient transfers etc. for talking about and reflecting on intraPC explicitly.
		*Operationalization*: *Explicitly implement a mindset for developing intraPC (awareness) and make sure that ‘learning intraPC’ is embedded in the workplace*.
	7	Supervisors themselves engage in intraPC as role models. By their own actions, supervisors can teach residents aspects of intraPC. Aware of the residents' work contexts, supervisors should stimulate residents to engage in intraPC.
		*Operationalization*: *Provide exposure to intraPC learning activities in placement workplaces*. *Trainers/supervisors are active role models for intraPC*. *SC trainers/supervisors are aware of PC residents' work contexts*. *Trainers/supervisors have the knowledge, skills and attitudes to coach residents in intraPC and connect with both contexts*.
	8	The training team engages explicitly in intraPC with the aim of delivering quality patient care and achieving continuous quality improvement.
		*Operationalization*: *The training team regularly reflects on its own intraPC approach and its effect on care and undertakes to work on areas for improvement* *(case discussion, 360 degree feedback, patient satisfaction, discussion of complications, feedback to residents upon placement completion)*.
	9	Bodies responsible for specialty programme goals define intraPC as a competency that *every* doctor should have. Formalise competencies and attainment targets relating to intraPC in the national, local and individual training plans of all specialisations.
		*Operationalization*: *Pay explicit attention to intraPC by PC and SC residents (in the workplace, the educational institution, the curriculum and peer groups on release days)*. *Focus on purposely intraPC learning (placement host)* *Facilitate getting to know each other's expertise and roles and ways of collaboration (placement host and curriculum)*.
	10	Supervisors, teachers and residents work to ensure that every resident knows how to engage in intraPC upon completion of their training. Regular discussion and assessment of residents' intraPC progress by supervisors.
		*Operationalization*: *Supervisors and residents utilise scheduled training meetings and assessments to discuss and evaluate intraPC*.
	11	Residents transfer intraPC lessons and apply them in their own work contexts. SC supervisors and PC teachers encourage Pc and SC residents during placements to discuss how intraPC lessons can be translated, transferred, transformed and integrated into their own work activities.
		*Operationalization*: *Facilitate conversations between PC and SC residents as well as between each of these groups with their peers*. *Connect both contexts by making explicit links between residents' placement experiences and their own work contexts in PC and SC settings*.

*Note*: Final set of 12 Design Principles for learning intraPC during hospital placements categorised into three clusters, entitled: **Culture** (Zero, 1, 2), **Connecting Contexts** (3, 4) and **Making the Implicit Explicit** (5–11). The Design‐Principles consist of two parts: (i) a title, describing the design principle (the dot on the horizon) and a subtitle, describing how the Design‐Principles aim can be achieved; (ii) an operationalization, describing what could be done to achieve the Design‐Principles aim.

### 3.2 The Culture cluster

The Culture cluster included three Design‐Principles (zero, 1, 2) that focused on the central role of the patient (zero) and on building collaborative relations based on equity between PC and SC physicians (2) and on building a safe learning environment where traditional power and culture differences between PC and SC physicians can be discussed (3). All FG participants agreed that Design‐Principle**‐zero** should be the starting‐point of intraPC learning.

‘The Design‐Principles should start with Design‐Principle‐zero such as “this is about good care for patients”. To get an SC physician on board, the patient needs to be prominently positioned in the Design‐Principles, I think. […] The patient is involved in everything we do: it's all about the patient, and we will use these design principles for the benefit of patient care, so the patient should be the foundation’. **SC supervisor_FG2**

Design‐Principle‐2 was initially formulated as ‘There is a safe learning environment where culture and power differences can be discussed’. During all three focus group sessions, several SC participants initially commented that it was unnecessary or too severe to include power differences in the Design‐Principles because, in their view, there were no power differences at play, only cultural differences. The PC participants, however, explicitly mentioned that they did experience power differences between PC and SC on a regularly basis. During the discussions in all FGs, participants unanimously agreed that Design‐Principle‐2 should focus on equity in working relations. The original Design‐Principle‐2 was finally adapted and entitled: ***Apply the principle that, in a intraPC partnership, we are all different but operate on a basis of equity*** and sub‐titled: ‘Supervisors and residents create a safe learning and working environment in which culture, equity and differences in work relations can be discussed’. The operationalisation was finetuned by FG3, focusing on a safe working/learning climate, respect and the recognition and discussion of power/cultural differences.

One SC resident said, “‛Power differences’ sounds very weighty to me. I think it's enough just to mention cultural differences.” An SC supervisor nodded in agreement, whereas a PC supervisor and resident both rose in their chairs and responded with disapproval. **Fieldnote_FG1**

‘There are definitely certain power relations at play’. **PC resident_FG2**

‘I notice that many PC physicians and residents struggle with the power differences with [SC physicians in] the hospital’. **PC supervisor_FG2**

‘Of course, there's a lot of complaining about primary care, like “they are all dullards”. I think it's not very conducive if you hear that every day. […] It's about respect, appreciation and equality’ **SC resident_FG3**

‘Could we then just call it differences [...] we are all different yet equal in collaboration’ **SC supervisor_FG2**

Participants also emphasised the importance of building relations in the Design‐Principles (1), as this was vital to establishing equal and mutual intraPC.

‘Building relations is important, but this is sometimes avoided in the hospital. Without building relations, there can be no [equal] collaboration, but rather one‐way cooperation with someone wanting something and the other person having to do it’. **SC resident1_FG3**

‘First build relations, and then make sure that we work with each other on an equal basis’ **SC resident2_FG3**

### 3.3 The connecting contexts cluster

The Connecting Contexts cluster includes two Design‐Principles (3, 4) that involve connecting and aligning primary and secondary care by mutual learning and collaboration between PC and SC residents and supervisors (3) and by acquiring knowledge of each other's work contexts and activities (4). FG participants noted that mutually sharing each other's contexts and activities was essential to learning how to align PC and SC and provide continuity of care. FG1 formulated a new Design‐Principle (**4**), which was further refined by FG2 and FG3 into: ***Facilitate the acquisition of knowledge of one another's work contexts and activities to promote good collaboration*.**

‘I'm in favour of mutual exchange [of placements] because then you [SC residents] also know where your patients are going to and coming from and how that [referral] goes’. **PC resident_FG1**

‘When I've seen a patient in the hospital and want to transfer him properly to primary care, what exactly does a PC physician need to know from me [SC physician] in order to continue to properly manage care? This is something I'd like to know’ **SC resident_FG2**

During work‐conference‐2, many participants identified referral and discharge letters as a useful opportunity for intraPC educational activities, See Box 2.

**BOX 2:** Prototype of educational activities for intraPC learning developed based on the DPrins during work conference 2

**Title education**	Learning from referral to and discharge from the hospital
**Education goals**	Sharing and getting to know each other's perspective on 1. Discharge from ward or outpatient department to home or nursing home 2. Referral from primary care to hospital Being able to write appropriate referral and discharge letters with knowledge of the different perspectives (PC and SC physicians)
**Live, online, hybrid**	Live at the hospital ward during daily work or education session
**Participants**	PC residents, SC residents, SC supervisors
**Preparation for participants**	Every participant selects a referral and/or a discharge letter and bring these anonymized letters to the joint discussion session.
**Practicalities**	Allocated time: e.g. 30–45 minutes a month during workplace learning or during an educational session in the ward.
**Method**	PC and SC residents and supervisors discuss referral letters and discharge letters. For example, 2–3 referral letters and 2–3 discharge letters during a session. Start: Present a patient case and read the letter. Dialogue: Based on the letter, participants discuss the goals of the referral and discharge letter, participants give each other feedback and share their perspectives. For example, referral: Is the referral question clear and is the referring perspective clear? For example, discharge: Is the question of the PC physician addressed properly in the discharge letter? Do the treatment recommendations fit the PC context? Debriefing: what would you do differently after this discussion.
**Design Principles**	0, 3, 4, 5, 6, 8

### 3.4 The Making the Implicit Explicit cluster

The Making the Implicit Explicit cluster included seven Design‐Principles (5–11) that involved interventions for intraPC learning both on the job and off the job explicit and intentional: paying deliberate attention to different perspectives (5) and intraPC during work activities (9), the encouragement of a ‘practise what you preach’ role model function from supervisors and the supervising team by demonstrating and continue advancing intraPC (7, 8), setting intraPC learning goals and competency profiles (9) and evaluations and assessments of intraPC during daily work (10, 11).

On Design‐Principle‐5 (***Collaborate on patients and pay deliberate attention to two‐way learning from different perspectives***), the FG3 participants discussed sharing professional expertise, emphasising the importance of proactively contributing PC knowledge during hospital placements to make SC physicians and residents aware of the possibilities and impossibilities in the PC setting.

‘SC physicians do not know very well what the struggles or impossibilities are in primary care. This is also where the comments arise [by SC physicians about PC physicians]. I would say PC residents bring PC knowledge and experience into the secondary care setting, structurally’. **SC supervisor_FG3**

Design‐Principle‐10 (***Work to ensure that every resident knows how to engage in intraPC upon completion of their training***) indicated that intraPC should be assessed as an important competency in various activities in the workplace. Residents in FG3 mentioned, however, that self‐assessments are likely to produce socially desirable answers that, hence, will fail to achieve their purpose. It is important for intraPC assessment to be linked to the existing assessment policies and tools in the training programme, by discussing and evaluating intraPC during regular supervision meetings, for example.

‘Testing and assessing intraPC is difficult, I think. If we can fill in the questionnaire with socially desirable answers, that's a risk. To me, talking and learning about intraPC is more important than us going back to filling out assessments because that will fail to achieve the goal’. **SC residents_FG3**

In order to facilitate intraPC learning among residents, the FG1 participants noted that the supervising team should also keep training themselves in intraPC based on the DPs. Therefore, a new Design‐Principle (**8**) was added *(**The training team engages explicitly in intraPC with the aim of delivering quality patient care and achieving continuous quality improvement**)*. FG3 participants, furthermore, noted that the supervising team should reflect on both the process and the outcome of intraPC.

‘You can only teach residents about intraPC if we ourselves, as a supervising team, also work and collaborate as an interprofessional team according to certain principles. Before we facilitated intraPC learning in our department, we first reflected in our team “how do we collaborate [with primary care] as a department, what goes well, what improvements are needed and how are we going to work on/achieve that.”’ **SC Supervisor_FG1**

In the next round, FG2 participants indicated the importance of having role models: individual supervisors demonstrating intraPC as physicians and departmental teams demonstrating continuous development in intraPC as a team. Since supervisors themselves may not yet be so adept at intraPC, FG participants emphasised that there should be space for supervisors to continue to learn intraPC themselves.

‘“Supervisors can teach residents aspects of intraPC based on their own actions, ” I like that. You rely on supervisors who are intraPC collaborators themselves. And that [doing intraPC] is the starting‐point for teaching other people. These Design‐Principles do not say that supervisors have to do it all perfectly, but it's just a starting‐point to talk about intraPC with residents’. **Educationalist_FG2**

### 3.5 Design‐Principles relevancy and applicability

Online prioritisation with Mentimeter© and online poll quotes resulted in quantitative data consisting of individual dichotomous prioritisation of the Design‐Principles and lists of 10‐point scales.

Both the focus group discussions and the online prioritisation surveys revealed that the participants unanimously agreed that the Design‐Principles belonging to the *Culture* theme (**zero, 1, 2**) are ‘must haves’ and should be considered as prerequisites for successful intraPC learning. Regarding the Design‐Principles in the *Connecting Contexts* cluster and the *Making the Implicit Explicit* cluster, participants differed in their prioritisation, which depended strongly on the pre‐existing workplace conditions.

The online poll quotes using a 10‐point scale (1–10) to check the feasibility and applicability of the Design‐Principles resulted in the following mean scores: (I) ‘The Design‐Principles are feasible for designing intraPC educational practice’, mean score: 7.2. (II) ‘The Design‐Principles are clearly formulated’, mean score: 7.6. (III) ‘The Design‐Principles are applicable in my daily work’, mean score: 7.3.

Some educationalists and policymakers mentioned that they do not design their own education, but that the Design‐Principles are nevertheless useful for them to verify whether intraPC educational activities meet relevant characteristics.

‘the Design‐Principles help to reflect on whether all essential characteristics have been addressed’. **Policy‐maker_WC2**

## 4 DISCUSSION

In this study, we developed a set of 12 theory‐driven and context‐sensitive Design‐Principles for learning intraPC between PC and SC residents during hospital placements. The Design‐Principles were categorised into three clusters: *Culture*, *Connecting Contexts* and *Making the Implicit Explicit*. The *Culture* cluster focuses on building relations based on equity allowing space to openly discuss traditional power dynamics and cultural differences between PC and SC physicians. The *Connecting Contexts* cluster focuses on connecting primary and secondary care and having PC and SC residents understand each other's work contexts and activities. The *Making the Implicit Explicit* cluster focuses on residents deliberately paying attention to intraPC learning on the job and off the job, and on having supervisors demonstrate and continually advance intraPC, also known as ‘practise what you preach’.

In a prior study, Kilty et al. described essential baseline conditions for learning in a clinical environment during postgraduate training.^51^ Our study provides a valuable complement to this study by providing Design‐Principles specifically aimed at designing intraPC learning between PC and SC residents during hospital placements. Our findings on the importance of a safe culture to enable intraPC learning is in line with prior studies.^51^, ^52^ With the Design‐Principles in the *Culture* cluster, moreover, we have formalised the creation of a culture of equal collaboration and learning in which power dynamics between PC and SC physicians can be discussed. Our study revealed that *Culture* cluster Design‐Principles are prerequisites for intraPC learning in hospitals.

### 4.1 Power dynamics

Throughout the development of our Design‐Principles, the topic of power dynamics emerged strongly and was consequently embedded in the final set of Design‐Principles. Power dynamics are often present in education and interprofessional collaboration^53^, ^63^, ^64^ in PC residents' hospital placements^19^, ^65^, ^66^ and can demotivate residents.^67^ Nonetheless, minimal attention has been given to these dynamics in medical education research.^19^, ^53^, ^68^ As a result, power is underexposed when developing educational activities.^53^ In Design‐Principle‐2, power differences are addressed on both levels: differences between PC and SC physicians and those between residents and supervisors. Power dynamics between PC and SC physicians persist tacitly, with PC often seen as having a lower status.^16^, ^19^ During all focus group sessions, power dynamics and the imbalances in their impact were confirmed when SC residents and supervisors opted to remove ‘power differences’ from the Design‐Principles (Design‐Principle‐2) because they felt that these were merely cultural differences. The PC participants, however, explicitly mentioned that they often struggled with power differences with SC physicians. We hypothesise that these different experiences of power dynamics can be attributed to the difference in their impact: lower‐status individuals appear to be more troubled by power dynamics than higher‐status individuals. This complexity should be taken into account when designing intraPC educational activities, for example by recognising historical patterns and feelings of differences in power and culture and opening these up for discussion.

### 4.2 Mutual and transformative learning

Participants mentioned that alignment of PC and SC and improvement of intraPC can be achieved if both PC and SC physicians get to know each other's work contexts and activities. This can be facilitated by exchanging residents between each other's settings. Sampson^69^ already demonstrated that educational activities across PC and SC silos could be used to modify behaviour and increase understanding. Göbel et al.^70^ opted for feedback between PC and SC physicians through frequent meetings to support intraPC. These observations are affirmed and further developed in our study, particularly as formalised in Design‐Principle‐3: *‘Facilitate learning together by working together’* and Design‐Principle‐4: *‘Facilitate the acquisition of knowledge of one another's work contexts and activities to promote good collaboration*’.

After the hospital placements, PC and SC residents have to transfer acquired knowledge, skills and insights concerning intraPC into their own (or future) work context. From the boundary crossing theory perspective, this can add to transformative learning, with both parties creating new ways of working in connection with each other.^71^ Transformative learning requires that members of two communities of practice work and learn together. This could be complex during hospital placements as only PC residents cross boundaries of their own practice into a new community of practice, SC settings, often resulting in unidirectional learning. PC residents learn predominantly unidirectionally from SC residents and supervisors.^27^ For transformative learning to take place, transfer to the own community of practice is required. Design‐Principle‐11 was developed to bridge both communities of practice and promote transformative learning. In Design‐Principle‐11, we formalised mutual transformative learning by having regular discussions facilitated by SC supervisors and PC teachers. During educational activities, these discussions could explicitly address the factors that influence transformation leading to profound changes in intraPC or new jointly constructed intraPC practice.

### 4.3 Practise what you preach supervisor (team)

Participants in our study called for an active role of the entire supervising team in demonstrating and providing intraPC learning. This is in line with theories of workplace learning.^72^, ^73^ Workplace learning processes are mostly unintentional, spontaneous and happening more or less unconsciously as a result of residents' daily work activities, rather than as a results of highly structured teaching programmes.^72^ We speak of professional learning in the workplace when spontaneous and often unconscious learning processes are connected to conscious reflection and interaction.^73^ Hospital placements are a special kind of workplace learning. For a long time, physicians were trained by way of apprenticeship models, granting residents legitimate entry into a community of practice.^74^ Our wider understanding of apprenticeship has recently undergone a change^75^: where the old apprenticeship models stressed immersion learning by simply gaining experience through exposure, new apprenticeship models stress that residents also learn from their role models how to think and reflect on the job. Supervisors, therefore, should take the lead as role models in intraPC and reflect on their own performance as a team and residents themselves should also play an active role in facilitating their own intraPC learning process.

### 4.4 Implications for practice and future research

We have chosen to use Design‐Principles as a guideline, a heuristic, which is a commonly used definition.^76^, ^77^ As Bakker^76^ describes it, this is ‘something to consider and try out, with the common sense understanding that no two situations will be identical and that adaptation to local circumstances is always necessary’.^76^ ^(p.52)^ This means that Design‐Principles should not be taken as prescriptions, but rather as guidelines that are meant to be achieved in a particular setting, supported by goals.^76^, ^77^ Our theory‐driven and context‐sensitive Design‐Principles were developed to guide the design of intraPC education between PC and SC residents during hospital placements, but we believe they could be adjustable in other contexts as well. Although participants of our work conferences found the Design‐Principles clear an feasible for designing intraPC educational activities, our study was conducted as prototyping phase in Design‐Based Research. Future research could further assess applicability of the Design‐Principles in educational practice in order to complete the Design‐Based Research approach.

Off course, the Design Principles will be assessed in phase III (assessment phase) of the Design‐Based Research. In a next study, we will investigate the educational interventions based on the principles. Beyond that, these Design‐Principles can to be taken into account in the reflection and feedback cycles when assessing residents. For instance, including patients and caregivers in providing feedback to residents, how patient‐centred care was provided by the resident, explicitly indicate, ask for and discuss cultural aspects of intraPC experienced by the resident, explicitly ask for learning from mistakes, utilise scheduled training meetings and assessments to discuss and evaluate intraPC.

By working with stakeholders, we were able to verify that the Design‐Principles are attractive and user‐friendly to those who have to work with them. In this regard, it is important to be aware of certain language used in Design‐Principles. As Cahn^78^ argues, curriculum developers often intend to create education with conceptual and logistical barriers in mind but tend to overlook the semantic element of language.^78^ Certain words could (un)consciously send messages that undermine the value of specific team members. This could expose any power dynamics even more explicitly and take the focus away from the collaboration^78^ one is striving to improve. The importance of language and nuance emerged during our study, as participants paid explicit attention to the wording of sentences and the description of words. As one FG2 participant said, ‘I like that, it's very much about language. That's actually at the basis of everything we do, to come up with a new common language that everybody understands’.

We think that the description of the development of Design Principles, together with stakeholders, researchers and patients/caregivers, provides a demonstration of a method that could be used for approaching complex educational challenges. As such, the design principles themselves could be used to guide intraPC educational activities. Furthermore, the description of developing these principles could be used as a method for approaching educational challenges such as enhancing collaboration between physicians.

### 4.5 Strengths and limitations of the study

A strength of this study is the start with solid theoretical data and the use of focus groups and work conferences, where rich and in‐depth data emerged from the interaction between participants from different areas of expertise and different communities of practice.^42^ Another strength is that this study focuses on both refining and testing context‐sensitive Design‐Principles and designing practical prototypes of activities in an iterative process.^36^, ^39^ In Design‐Based Research for education development, researchers often serve as developers of educational activities.^79^, ^80^, ^81^ Their active involvement in learning and teaching procedures, engaging with stakeholders, manifests scientific and educational value.^79^, ^80^ Furthermore, the process of developing Design‐Principles can also be informative. Another strength is the transferability of design principles to a wide range of hospital placements. Although postgraduate training varies considerably both within and across countries and cultures, there are also strong similarities: postgraduate training around the world is predominantly workplace‐based; residents in training for PC or SC physician undertake placements in their own specialty and additionally in other specialties. It is common worldwide for PC residents to spend a majority (months or years) of their training in the hospital.^30^, ^82^ Most training programmes for SC residents also consist largely of out‐of‐specialty placement in various hospital departments of other specialties worldwide.^83^, ^84^, ^85^, ^86^ During these placements, residents work with residents from other PC and SC specialties in the same hospital ward and have the opportunity to learn intraPC. Since these placements have similar practices, such as patient‐centred workplace learning, the existing power dynamics and cultural differences between specialties, the need to get to know and understand each other's work contexts and supervisors who continue to develop intraPC as role models, we think the Design‐Principles will be relevant to a broad range of international postgraduate training.

Our study also has limitations. This study was conducted in the Netherlands with reference to the Dutch postgraduate training programmes. Even though many countries operate similar hospital placement programmes and settings, we did not uncover global principles. We do, however, argue that the Design‐Principles may be adapted in countries where the placement setting is somewhat different. Every postgraduate training programme must, therefore, keep its own particularities in mind when implementing these Design‐Principles in its own setting and when evaluating their application. By providing rich context descriptions with our focus group sessions and work conferences and by including professionals and residents from different professional backgrounds as well as patients/caregivers, this study provides guidelines (Design‐Principles) that are transferable to a wide range of hospital placements or other medical workplace learning environments.^35^

## 5 CONCLUSION

To facilitate intraPC learning during hospital placements, designing activities on various levels is needed: (i) *Culture*: building collaboration based on equity in a psychologically safe learning/working environment where patterns or feelings of (in)equality, power dynamics and cultural differences can be discussed; (ii) *Connecting Contexts*: making residents and supervisors understand each other's work context and activities by mutual learning and exchanging residents in each other's settings; (iii) *Making the Implicit Explicit*: by consciously focusing on residents' intraPC learning and by having supervisors act as role models demonstrating intraPC and continuously pursuing intraPC improvement as a team.

## CONFLICT OF INTEREST

The authors declare no conflicts of interests.

## ETHICS STATEMENT

This study was approved by the Ethical Review Board (ERB) of the Dutch Organization for Medical Education NERB dossier number: 2020.1.4. Written informed consent for the use of the audio recordings and gathered data was obtained from the participants.

## AUTHOR CONTRIBUTIONS

Natasja Looman met all of the following conditions: substantial contributions to the conception and design of the work; the acquisition, analysis and interpretation of data for the work; AND drafting the work and revising it critically for important intellectual content; AND final approval of the version to be published; AND agreement to be accountable for all aspects of the work in ensuring that questions related to the accuracy or integrity of any part of the work are appropriately investigated and resolved.

Jacqueline de Graaf met all of the following conditions: substantial contributions to the conception and design of the work; the acquisition, analysis and interpretation of data for the work; AND drafting the work and revising it critically for important intellectual content; AND final approval of the version to be published; AND agreement to be accountable for all aspects of the work in ensuring that questions related to the accuracy or integrity of any part of the work are appropriately investigated and resolved.

Bart Thoonen met all of the following conditions: substantial contributions to the conception and design of the work; the acquisition, analysis and interpretation of data for the work; AND drafting the work and revising it critically for important intellectual content; AND final approval of the version to be published; AND agreement to be accountable for all aspects of the work in ensuring that questions related to the accuracy or integrity of any part of the work are appropriately investigated and resolved.

Dieneke van Asselt met all of the following conditions: substantial contributions to the acquisition, and interpretation of data for the work; AND revising the work critically for important intellectual content; AND final approval of the version to be published; AND agreement to be accountable for all aspects of the work in ensuring that questions related to the accuracy or integrity of any part of the work are appropriately investigated and resolved.

Esther de Groot met all of the following conditions: substantial contributions to the acquisition, and interpretation of data for the work; AND revising the work critically for important intellectual content; AND final approval of the version to be published; AND agreement to be accountable for all aspects of the work in ensuring that questions related to the accuracy or integrity of any part of the work are appropriately investigated and resolved.

Anneke Kramer met all of the following conditions: substantial contributions to the acquisition, and interpretation of data for the work; AND revising the work critically for important intellectual content; AND final approval of the version to be published; AND agreement to be accountable for all aspects of the work in ensuring that questions related to the accuracy or integrity of any part of the work are appropriately investigated and resolved.

Nynke Scherpbier met all of the following conditions: substantial contributions to the conception and design of the work; the acquisition, analysis and interpretation of data for the work; AND drafting the work and revising it critically for important intellectual content; AND final approval of the version to be published; AND agreement to be accountable for all aspects of the work in ensuring that questions related to the accuracy or integrity of any part of the work are appropriately investigated and resolved.

Cornelia Fluit met all of the following conditions: substantial contributions to the conception and design of the work; the acquisition, analysis and interpretation of data for the work; AND drafting the work and revising it critically for important intellectual content; AND final approval of the version to be published; AND agreement to be accountable for all aspects of the work in ensuring that questions related to the accuracy or integrity of any part of the work are appropriately investigated and resolved.

## Data Availability

The data that support the findings of this study are available from the corresponding author upon reasonable request.
